# Editorial: Apoplastic effectors — What roles do they play in plant-pathogen interactions?

**DOI:** 10.3389/fmicb.2023.1149771

**Published:** 2023-02-14

**Authors:** Xiao-Ren Chen, Yonglin Wang, Shiv D. Kale, Yufeng Fang, Vaibhav Srivastava

**Affiliations:** ^1^College of Plant Protection, Yangzhou University, Yangzhou, China; ^2^College of Forestry, Beijing Forestry University, Beijing, China; ^3^Fralin Life Science Institute, Virginia Tech, Blacksburg, VA, United States; ^4^GreenLight Biosciences Inc., Research Triangle Park, NC, United States; ^5^Division of Glycoscience, Department of Chemistry, School of Engineering Sciences in Chemistry, Biotechnology and Health, KTH Royal Institute of Technology, AlbaNova University Center, Stockholm, Sweden

**Keywords:** apoplastic effectors, plant pathogens, virulence function, PTI, interaction, evolution

Plant pathogens, such as oomycetes, fungi, and bacteria, are responsible for significant plant production losses worldwide in agriculture and natural ecosystems. These phytopathogens have evolved intricate mechanisms to facilitate their infections. It is widely recognized that phytopathogens employ an array of secreted proteins (called “effectors”) to manipulate plant physiological processes and defense systems in favor of their infection. Accordingly, plants have developed a sophisticated innate immune system to defend themselves against such attacks.

The apoplastic space between the plant cell wall and the plasma membrane constitutes the important battle frontline for plant-pathogen interactions. To survive in the harsh apoplastic environment, pathogens must cope with various immune responses. To do so, pathogens secrete an arsenal of apoplastic effectors into the apoplast milieu. Some of them were known to be detected by the plant surveillance system or to counterstrike plant immune responses; however, most apoplastic effectors' nature, functions, and roles during the interactions are still a conundrum. Recently, much attention has been paid to such effectors. Increasing evidence shows that apoplastic effectors play essential roles in plant-pathogen interactions.

In this context, we organized this Research Topic on “Apoplastic effectors—what roles do they play in plant-pathogen interactions?”. The topic collected six excellent papers (four research articles and two reviews) from international researchers and has already had over 7,200 views. The topic covers the following two themes:

## Characterization of apoplastic effectors

Phytopathogen-secreted effector proteins are essential in establishing a successful infection by modulating plant immunity. Studying effector proteins is important to know more about their roles in pathogen-host interactions.

*Cladosporium fulvum* causes leaf mold on tomato by secreting several effector proteins (i.e., extracellular proteins, Ecps) into the plant apoplast to suppress plant immunity. One of the genes, *Ecp20-2*, was characterized and functionally analyzed by Karimi-Jashni et al. using combined omics approaches including transcriptome, mass spectrometry and gene deletion analyses. It was revealed that this novel effector is required for full virulence of *C. fulvum* in tomato.

In contrast to *C. fulvum, Dothistroma septosporum* is a hemibiotrophic fungus whose effectors are poorly characterized. Tarallo et al. identified two apoplastic effector protein families, Ecp20 and Ecp32, in *D. septosporum* and analyzed their orthologs in *C. fulvum* as well. Their ability to trigger cell death in the non-host species *Nicotiana benthamiana* and *Nicotiana tabacum*, and in the gymnosperm host *Pinus radiate* was investigated. The work provides insights into the conservation of core effector function and recognition across a broad range of plant species.

Liu et al. reported the identification of 11 necrosis- and ethylene-inducing peptide 1-like proteins (NLPs) of *Colletotrichum australisinense* that cause rubber tree anthracnose. They studied in-depth the necrosis-inducing activity of selected NPLs belonging to three groups/types. The research demonstrates that CaNLPs are functionally and spatially distinct and may play different but important roles in *C. australisinense* pathogenesis.

He et al. discovered a secreted ribonuclease (SRE1) from *Setosphaeria turcica*, a fungus responsible for northern corn leaf blight disease. SRE1 and its homologs in other phytopathogens could induce cell death in plants, indicating their role in the fungal virulence. This study also clearly shows how pathogen effectors can activate plant immunity and enhance plant resistance to oomycetes and other fungal pathogens.

## Known and prospective roles of effectors

Plants can detect surrounding microbes in apoplast by recognizing microbe-associated molecular patterns (MAMPs) by pattern recognition receptors (PRRs) on the cell surface to activate appropriate signaling. In response, microbes have developed strategies to counteract this process. Lü et al. summarized recent findings on how the formation, concentration, and size of mature MAMPs affect the PRR-mediated immune signaling. In particular, the authors describe a few potential applications of effectors and explore open questions in the fields.

To establish successful infection, phytopathogenic microbes typically secrete many effectors either into the host apoplast or inside the cells to manipulate host immunity. Understanding their functions and roles may, in turn, shed light on developing new strategies against phytopathogenic fungi and oomycetes. To this end, Lu et al. summarized recent advances in genomic identification, transcriptional profiling, and functional characterization of proteinaceous effectors in pathogenic *Colletotrichum* fungi. In this review, Lu et al. gained novel insight about how effectors block fungal chitin-induced host signaling, suppress hypersensitive cell death and reactive oxygen species generation, and induce plant immunity. It would also be interesting to explore the underlying mechanisms of how *Colletotrichum* effectors are secreted and delivered during the pathogen–plant interactions.

The research progress reported in this Research Topic provides key insights into the apoplastic effectors of microbes. It expands our knowledge toward understanding the molecular interactions between host plants and phytopathogens in the apoplast. It will eventually facilitate the development of novel disease control strategies.

## Author contributions

All authors listed have made a substantial, direct, and intellectual contribution to the work and approved it for publication.

